# Highly pathogenic avian influenza A H5N1 virus infection in an immunocompromised domestic cat

**DOI:** 10.1128/asmcr.00134-25

**Published:** 2025-09-12

**Authors:** Chi Chen, Akhila Naru, Vineetha Reddy Mareddy, Saraswathi Lanka, Colleen Olmstead, Vanessa Revindran-Stam, Megan Sherman, Heather Yee, Natara Loose, Martha A. Delaney, Miranda D. Vieson, Ying Fang

**Affiliations:** 1 Department of Pathobiology, College of Veterinary Medicine, University of Illinois at Urbana-Champaign, Urbana, Illinois, USA; 2 Veterinary Diagnostic Laboratory, College of Veterinary Medicine, University of Illinois at Urbana-Champaign 14589, Urbana, Illinois, USA; 3 The Neighborhood Vet, Brooklyn, New York, USA; 4 Zoological Pathology Program, University of Illinois, Brookfield, Illinois, USA; Rush University Medical Center, Chicago, Illinois, USA

**Keywords:** highly pathogenic avian influenza (HPAI), H5N1, feline, cat, feline infectious peritonitis, diabetes mellitus, whole-genome sequencing

## Abstract

**Background:**

Highly pathogenic avian influenza (HPAI) H5N1 viruses of clade 2.3.4.4b have recently caused widespread outbreaks in mammals, including domestic cats that live closely with humans and other animals. In-depth molecular and pathological characterizations of naturally infected cats are urgently needed for developing better strategies to prevent interspecies transmission and further spreading of these viruses.

**Case Summary:**

In this case report, we characterized a unique case of HPAI H5N1 virus infection in an immunocompromised domestic cat. The pet animal was a diabetic cat with a history of feline infectious peritonitis (FIP). In early 2025, the cat developed acute fever and rapidly worsening respiratory distress and liver dysfunction despite antibiotic treatment. Due to severe clinical deterioration, the cat was euthanized. Postmortem examination revealed severe bronchointerstitial pneumonia, hepatic and lymphoid necrosis, bone marrow degeneration, and mild lymphohistiocytic meningitis. H5N1 viral RNA/antigens were specifically detected in the lung, brain, urine, or lymphoid tissues. Whole-genome sequencing and phylogeny analysis identified that the virus belongs to influenza clade 2.3.4.4b (B3.13 subgroup), closely related to HPAI H5N1 strains that are currently circulating in domestic cats and cattle. The source of infection for this particular cat might be linked to a fomite/environmental transmission route.

**Conclusion:**

The lethal HPAI H5N1 virus infection in an immunocompromised cat highlights the need for developing an improved prevention plan for pet animals. Clinicians should consider the possibility of H5N1 virus infection in cats with similar acute respiratory or neurologic signs, particularly in animals with chronic illness.

## INTRODUCTION

Highly pathogenic avian influenza A H5N1 virus, a subtype originating from the A/goose/Guangdong/1/1996 H5 subtype, continues to circulate among wild and domestic birds and spillover to mammals (1). The Eurasian H5N1 clade 2.3.4.4b was first detected in North America in late 2021 and has since caused spillover infections and deaths in terrestrial and marine mammals across the United States (2–7). The detection of HPAI H5N1 clade 2.3.4.4b in severe human infections raises concern about its pandemic potential (8, 9). The recent detection of HPAI H5N1 virus in dairy cattle presents an unusual transmission route (10, 11). Notably, domestic cats have been involved in infections on dairy farms, where cats were exposed to H5N1 virus by ingesting raw milk from infected cows or through contact with contaminated farm environments (7, 12, 13). Cats were also infected by H5N1 virus through consuming infected birds (14–16). As a companion animal, cats may function as an intermediate host to facilitate the cross-species transmission of viruses between animals and humans. Domestic cats appeared to be highly susceptible to HPAI H5N1 virus and developed severe respiratory and neurological symptoms and, in some cases, resulted in the death of the animal (17, 18). In this study, we characterized a case of H5N1 virus infection in a diabetic cat with a history of FIP virus (FIPV) infection.

## CASE PRESENTATION

A 1-year-old male, castrated domestic shorthair cat was submitted to the University of Illinois (UIUC) Veterinary Diagnostic Laboratory in February 2025 for postmortem examination. This cat was owned and housed at the submitting veterinary clinic in Brooklyn, New York, and had a previous diagnosis of FIPV infection at 8 weeks of age, which was treated with 10 mg/kg/day GS-441524 (Lucky Cat Veterinary Care), initially with the injectable formulation and then switched to oral administration after a seroma developed at an injection site. After recovery from clinical signs attributed to FIP, the cat developed diabetes mellitus that was continually difficult to regulate, contributing to multiple episodes of diabetic ketoacidosis (DKA). During a period of diabetic regulation, the cat developed a high fever (105.7^o^F) with slightly different symptoms from previous DKA episodes, prompting emergency care where antimicrobial therapy (Unasyn and Baytril) and supportive care (IV fluids, ondansetron, insulin, metoclopramide, mirataz, and gabapentin) were initiated. Despite these efforts, the fever persisted, and the cat quickly progressed into respiratory and hepatic decline, followed by humane euthanasia.

**Fig 1:**
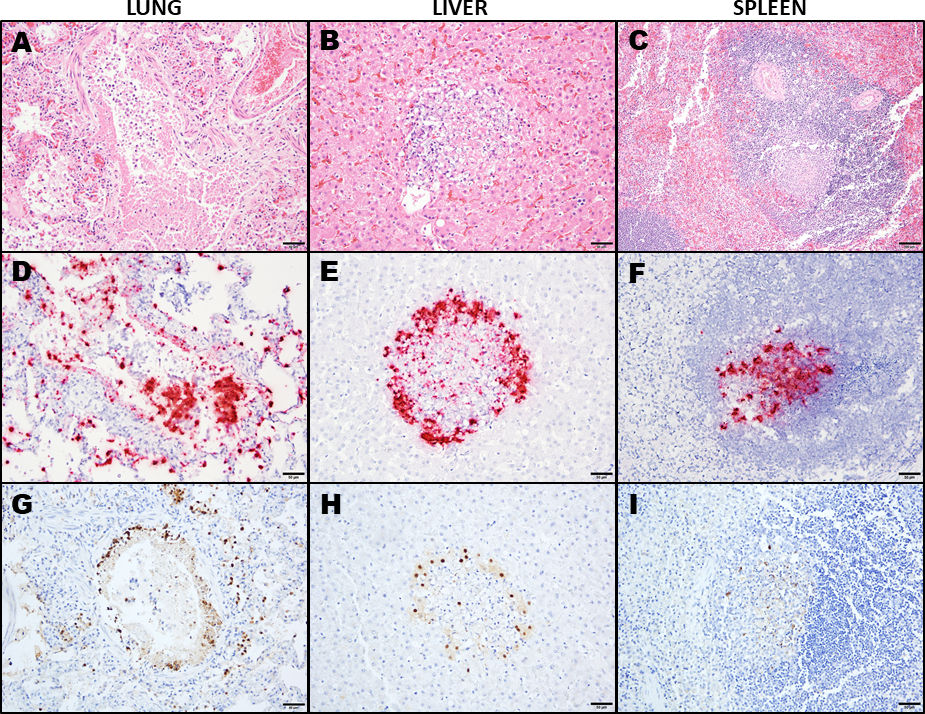
Photomicrographs of necrotizing lesions in a cat infected by the H5N1 influenza virus. Lesions include necrotizing bronchiolitis and bronchitis (**A, D and G**), hepatic necrosis (**B, E and H**), and splenic lymphoid necrosis (**C, F and I**) with colocalization of H5 RNA or influenza A virus NP antigen. (**A–C**) Hematoxylin and eosin staining; (**D–F**) RNAscope *in situ* hybridization with H5 RNA-specific probe; and (**G–I**): immunohistochemical staining with anti-NP monoclonal antibody (mAb #42-100).

Gross lesions were mild and non-specific, including multifocal mottling of the lungs, a few small fibrous adhesions between the omentum, spleen, and ventral internal body wall, and mild pallor and swelling of the liver. Significant histopathological lesions were found (Fig. 1A through C), including necrotizing bronchointerstitial pneumonia and fibrinosuppurative exudative interstitial pneumonia. Furthermore, there was necrotizing hepatitis, splenitis, lymphadenitis, bone marrow degeneration, and very mild multifocal lymphohistiocytic meningitis. To detect H5N1 infection, RNAscope *in situ* hybridization was performed using a probe for Hemagglutinin (HA) H5 gene region (ACD Diagnostics, Probe- V-InfluenzaA-H5N8-M2M1-C1 and RNAscope 2.5 HD Red Assay), while immunohistochemistry was conducted using monoclonal antibody (mAb #42-100) recognizing viral nucleoprotein (NP). The results showed intense H5 gene-specific signal amplification (Fig. 1D through F) and NP antigen-specific immunolabeling (Fig. 1G through I) within the areas that had significant lesions in the lung, liver, and spleen.

HPAI H5N1 infection was confirmed by RT-qPCR using the National Animal Health Laboratory Network approved protocol in UIUC Veterinary Diagnostic Laboratory, which targets Matrix (M) and H5 genes. The results revealed a high level of viral RNA load in the brain (M Ct = 17.13; H5 Ct = 19.16), lung (M Ct = 16.54; H5 Ct = 22.71), spleen (M Ct = 19.48; H5 Ct = 22.41), and urine (M Ct = 22.22; H5 Ct = 25.07). Further testing on cat tissue samples yielded negative results for FIPV, SARS-CoV-2, and *Francisella tularensis*. The aerobic culture of the lung and liver did not result in any significant pathogens, ruling out bacterial sepsis.

After the final diagnosis, contact tracing by the referring clinic revealed that a few days before the onset of illness, as a resident of the clinic, the cat had interacted with the clinic areas and personnel that had not yet been decontaminated from another feline patient. The patient presented with a painful abdomen, fever (103.9^o^F), and consumption of food that was recalled for the potential to contain H5N1 virus (19). There is no confirmation of H5N1 infection from that feline patient, and there were also no other known sources of exposure. No related human cases of H5N1 infection were identified.

**Fig 2:**
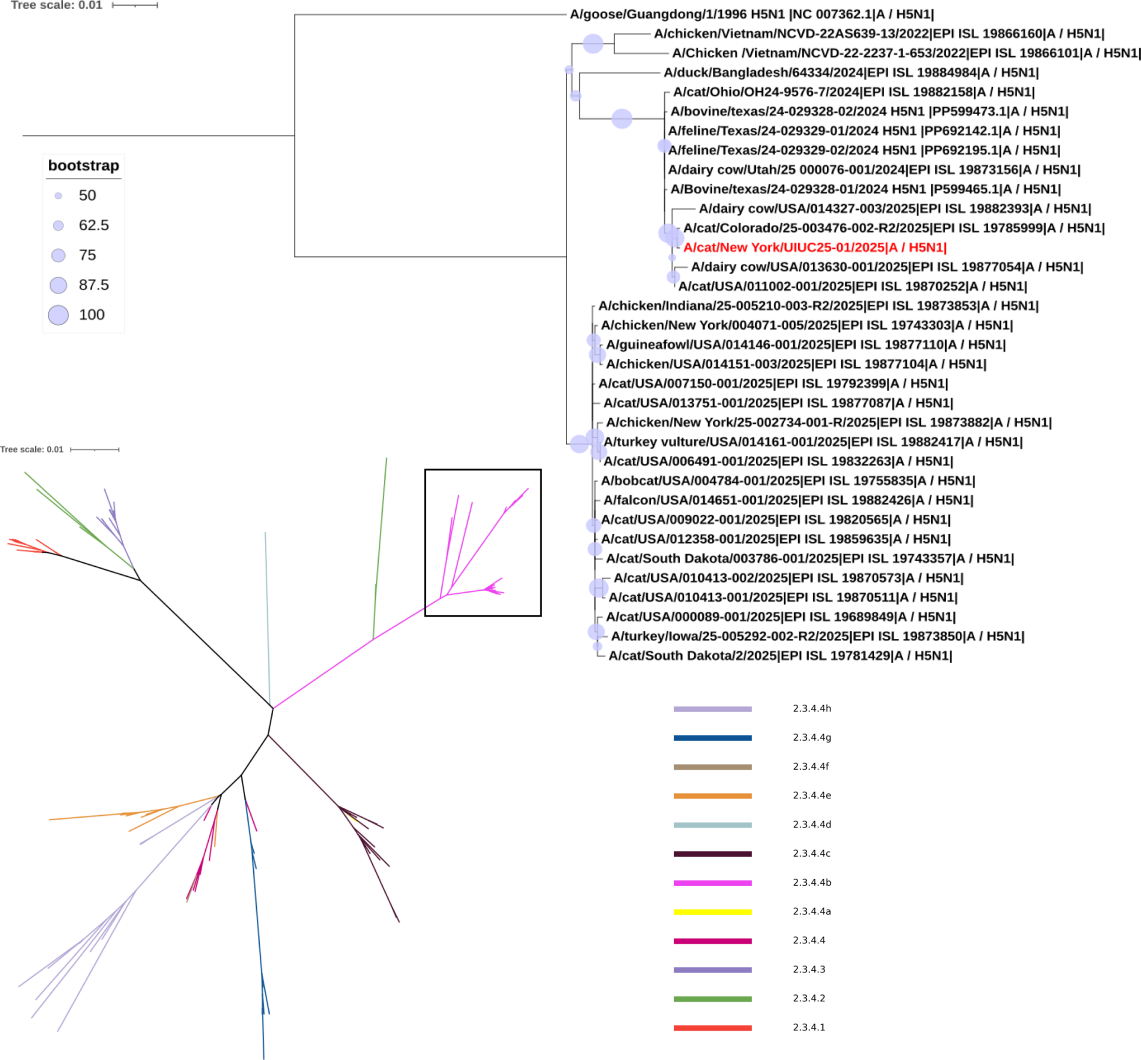
Phylogenetic analysis of hemagglutinin (HA) sequences. Full maximum likelihood tree of 117 HA sequences, color-coded by clade. Inset tree of clade 2.3.4.4b, rooted with *A/goose/Guangdong/1/1996_H5N1* (outgroup). The cat sequence analyzed in this study is highlighted in red. Bootstrap values ≥ 50% are shown at nodes (circles scaled by support: light to dark blue/purple = 50%–100%). Branch lengths reflect substitutions per site (scale bar). Trees were inferred using IQ-TREE (21) (v.2.2.6, GTR + F + G4 model, 1,000 UFBoot replicates) and visualized in the Interactive Tree of Life (iTOL) (22). GenBank no. NC007362.1 (outgroup), no. OF011022.1 for 2.3.4.4 a, no. PP599465.1 and no. PP599473.1 for 2.3.4.4b, and no. OF011048.1 for 2.3.4.4d were downloaded from NCBI GenBank. The rest of the sequences were from GISAID (23). Sequences were aligned using MAFFT (v7.526) (24).

**Fig 3:**
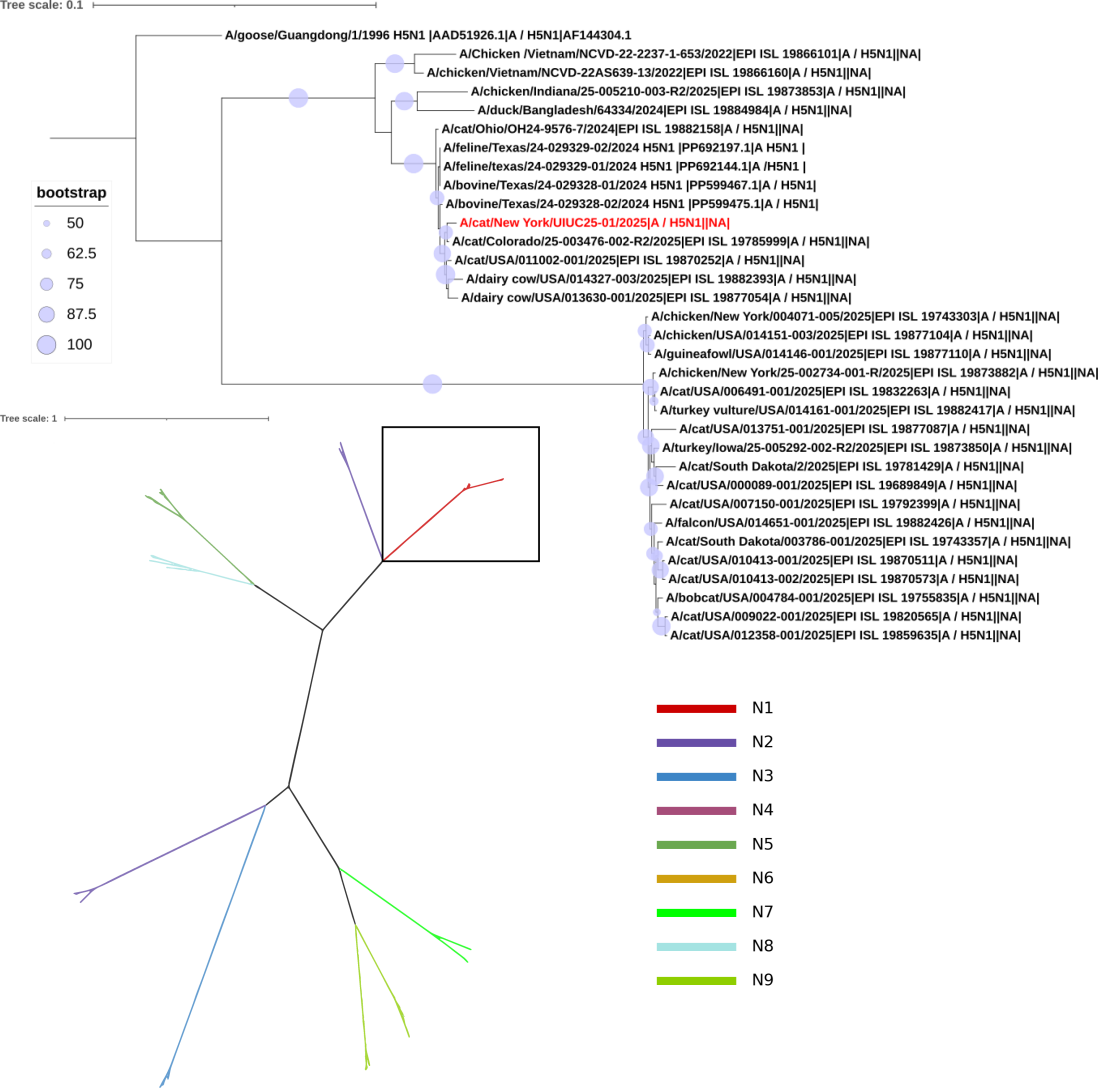
Phylogenetic analysis of neuraminidase (NA) sequences. Maximum likelihood tree of 95 NA sequences representing subtypes N1–N9, color-coded by subtype. Inset tree of N1 subtype sequences, rooted with *A/goose/Guangdong/1/1996_H5N1* (outgroup). The cat sequence analyzed in this study is highlighted in red. Bootstrap values ≥ 50% are shown at nodes (circles scaled by support: light to dark blue/purple = 50%–100%). Branch lengths reflect substitutions per site (scale bar). Trees were inferred using IQ-TREE (21) (v.2.2.6, GTR + F + G4 model, 1,000 UFBoot replicates) and visualized in the Interactive Tree of Life (iTOL) (22). GenBank no. AF144304.1 (outgroup) and no. PP599467.1, no. PP599475.1, no. PP692144.1, and no. PP692197.1 for N1 clade were downloaded from NCBI GenBank. The rest of the sequences were from GISAID (23). Sequences were aligned using MAFFT (v7.526) (24).

Viral whole-genome sequencing for brain and lung tissue samples was performed using Illumina MiSeq (20). The resulting sequences were identified as HPAI H5N1 virus genome segments. The cat-derived virus strain is designated as A/cat/New York/UIUC25-01/2025 (UIUC25-01). Subsequently, maximum likelihood phylogenetic trees were constructed using IQ-TREE (v2.2.6) (21). HA (Fig. 2) and NA (Fig. 3) phylogenies confirm that the UIUC25-01 virus belongs to 2.3.4.4b clade with other circulating HPAI H5N1 strains. Compared to the emerging H5N1 strain originally obtained from the A/Bovine/texas/24-029328-01/2024 (H5N1), the UIUC25-01 virus exhibited high genetic similarity across all genome segments (99.52%–99.86% nucleotide identity, 99.13%–100% amino acid identity; Table 1). One distinct mutation at amino acid position 71 of the NA protein (N71S) was identified in the cat virus, which was previously reported as a hallmark of emerging HPAI H5N1 viruses (2).

**TABLE 1:** Genome sequence comparison of cat virus UIUC25-01 with bovine virus A/Bovine/texas/24-029328-01/2024 (H5N1)

Segment	GenBank accession no.^*a*^	% nucleotide identity	% amino acid identity
HA	PP599465	99.61	99.47
NA	PP599467	99.52	100
PB1	PP599463	99.86	100
PB2	PP599462	99.70	99.74
NP	PP599466	99.55	99.40
NS	PP599469	99.66	99.13
M	PP599468	99.81	99.60
PA	PP599464	99.77	99.72

^*a*^ GenBank accession numbers listed in the table refer to the A/Bovine/texas/24-029328-01/2024 (H5N1) virus.

## DISCUSSION

In this report, we characterized a clinical case of HPAI H5N1 virus infection in a 1-year-old immunocompromised cat. Pathological and molecular analyses showed that the lung appeared to be the primary site of viral replication with variable degrees of systemic tissue damage. Consistent with the respiratory and hepatic decline reported clinically, the most severe pathology was observed in the lung and liver, with virus identified by immunohistochemistry and *in situ* hybridization, along with the necrotizing inflammation. Lesions, organs affected, and viral distribution in this case are similar to those previously documented in cats infected with HPAI H5N1 viruses (25, 26). Multiple cases have similarly shown higher levels of virus in the brain, often with lesions that are equally or more severe than those in the respiratory system, indicating a shift towards neurotropism (2, 10). Interestingly, in this case, there was minimal multifocal lymphohistiocytic inflammation in the leptomeninges and no neurologic symptoms mentioned in the clinical history. Despite this, using an H5-specific probe, RT-qPCR detected the highest level of viral RNA in brain tissue compared to that detected in the lung and spleen. This disparity may be related to spatial distribution. Assuming viral entry into the CNS through the olfactory nerves or across the cribriform plate, as has been documented in mice (27), more virus would be located in the frontal and olfactory regions of the brain that were submitted for testing but not examined by histopathology. Still, this specific route of viral entry into the brain of cats is not clearly documented (25) and warrants further investigation.

The exact source of infection for this cat has not been confirmed. Given no evidence of dairy or poultry consumption or contact with birds, cattle, or individuals working in poultry or cattle industries, we suspect that this cat may have been infected by fomite/aerosol transmission in the clinic by another cat with possible H5N1 virus infection. This highlights the importance of hand hygiene/decontamination between patients for healthcare. Another important consideration in this cat is concurrent diabetes mellitus and prior infection with FIPV, both of which can be related to a weakened immune system that may promote transmission of the H5N1 virus. This indicates that animal health conditions need to be considered when assessing spillover risk. Animal handlers/healthcare providers should understand the potential risks for immunocompromised animals sharing the same space with other infected animals.

In comparison to the emerging bovine-derived H5N1 virus A/Bovine/Texas/24-029328-01/2024, the cat UIUC25-01 virus shared >99% identity at the nucleotide and amino acid levels, indicating the same origin. Phylogenetic analysis further confirmed that this UIUC25-01 virus lies in the North American clade 2.3.4.4b, where it clusters with other recently reported emerging HPAI H5N1 strains from cats and dairy cows. Several mutations had been acquired by clade 2.3.4.4b viruses to adapt to mammalian hosts, particularly in cattle, marine mammals, and humans in recent outbreaks (2, 28–32). In comparison to our cat H5N1 virus sequences, the previously reported N71S mutation was found in the NA gene (2). This mutation is also present in several other H5N1 virus isolates from both cattle and cats (2, 30). The N71S mutation is located in the stalk region between the catalytic head and the transmembrane domain of NA. Although it is far from the catalytic site, unlikely to directly affect substrate specificity, this mutation may introduce a new phosphorylation or glycosylation site that may increase stalk rigidity and indirectly decrease neuraminidase-mediated virion release (33, 34). Functional characterization is warranted to determine how N71S affects NA enzymatic activities and overall viral fitness.

In conclusion, we have identified HPAI H5N1 virus infection in a domestic pet cat with chronic illness and performed molecular and pathological analyses. Our study suggests that the immunocompromised patients and their environmental/fomite contacts in clinical settings should be closely monitored to reduce the risk of cat-to-cat and cat-to-human transmission.

## Data Availability

The complete genome sequence of the H5N1 strain identified in this case has been deposited in GenBank under accession numbers PV780461, PV780462, PV780463, PV780464, PV780465, PV780466, PV780467, and PV780468. Additional metadata are available upon request.
